# The "Royal" Medical Benevolent Fund

**Published:** 1912-08-10

**Authors:** Samuel West

**Affiliations:** Hon. Treasurer. 11 Chandos Street, Cavendish Square, London, W.


*To the Editor of* The Hospital.

### The "Royal" Medical Benevolent Fund.

*To the Editor of* The Hospital.

Sir,—His Majesty the King has been graciously pleased to grant to the British Medical Benevolent Fund the title of Royal, so that it will henceforth be known as the Royal Medical Benevolent Fund. I shall be much obliged if you will assist in making known this act of the King's grace towards this long-established and well-deserving charity.—I am, Sir, your faithfully,

Samuel West, Hon. Treasurer.

11 Chandos Street, Cavendish Square, London, W.

